# Supplementary figures and images for: Patient Stratification of Clear Cell Renal Cell Carcinoma Using the Global Transcription Factor Activity Landscape Derived From RNA-Seq Data
Source: Front Oncol. 2020 Dec 4;10:526577. doi: 10.3389/fonc.2020.526577 (PMC7746882; doi:10.3389/fonc.2020.526577)

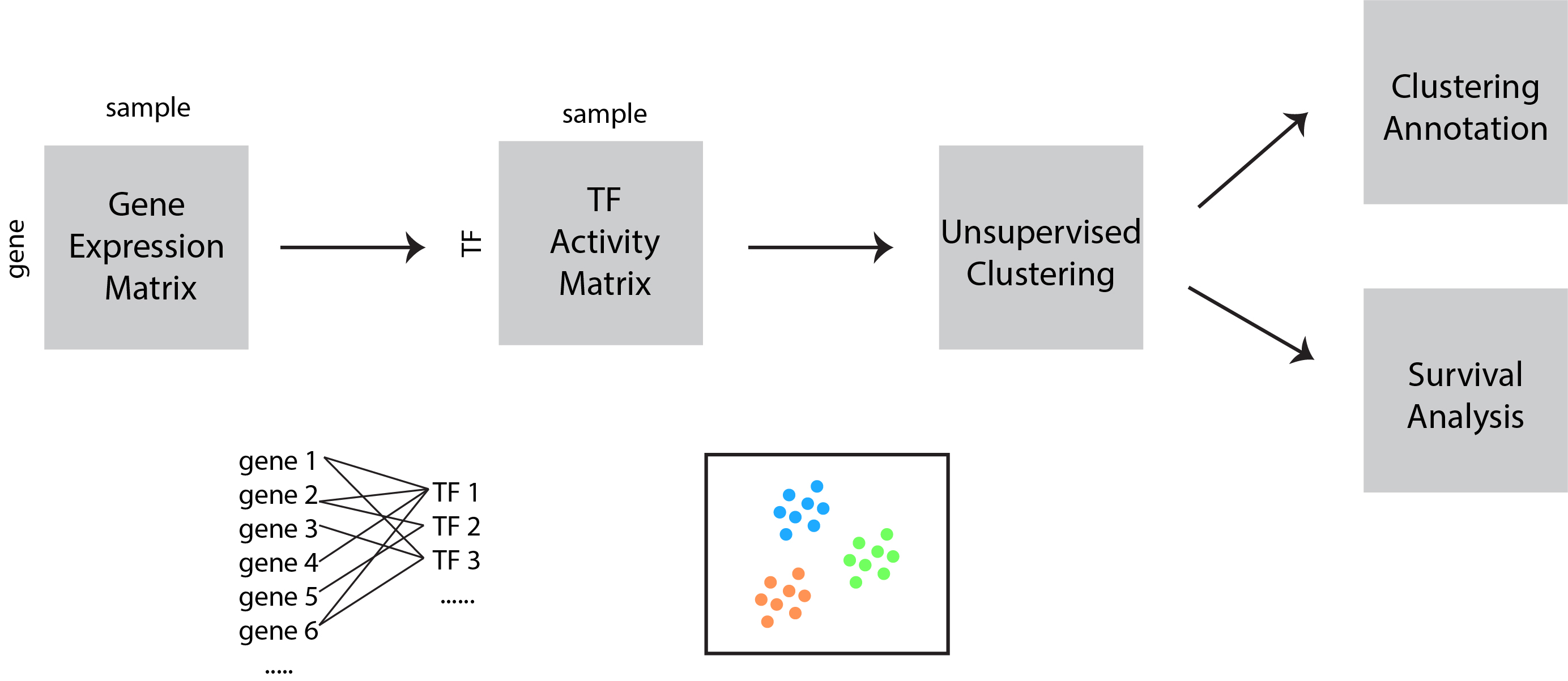

Supplement: Supplementary Figure 1 — Workflow illustrating the analysis pipeline undertaken. Gene expression matrix was projected to transcription factor activity matrix, which was used as input for unsupervised clustering. Cluster labels were used for patient stratification. Different clusters were annotated using the differentially expressed genes as compared to the rest of clusters. Survival analysis was performed to obtain an overview of the OS of distinct patient populations. [file Image_1.jpg]

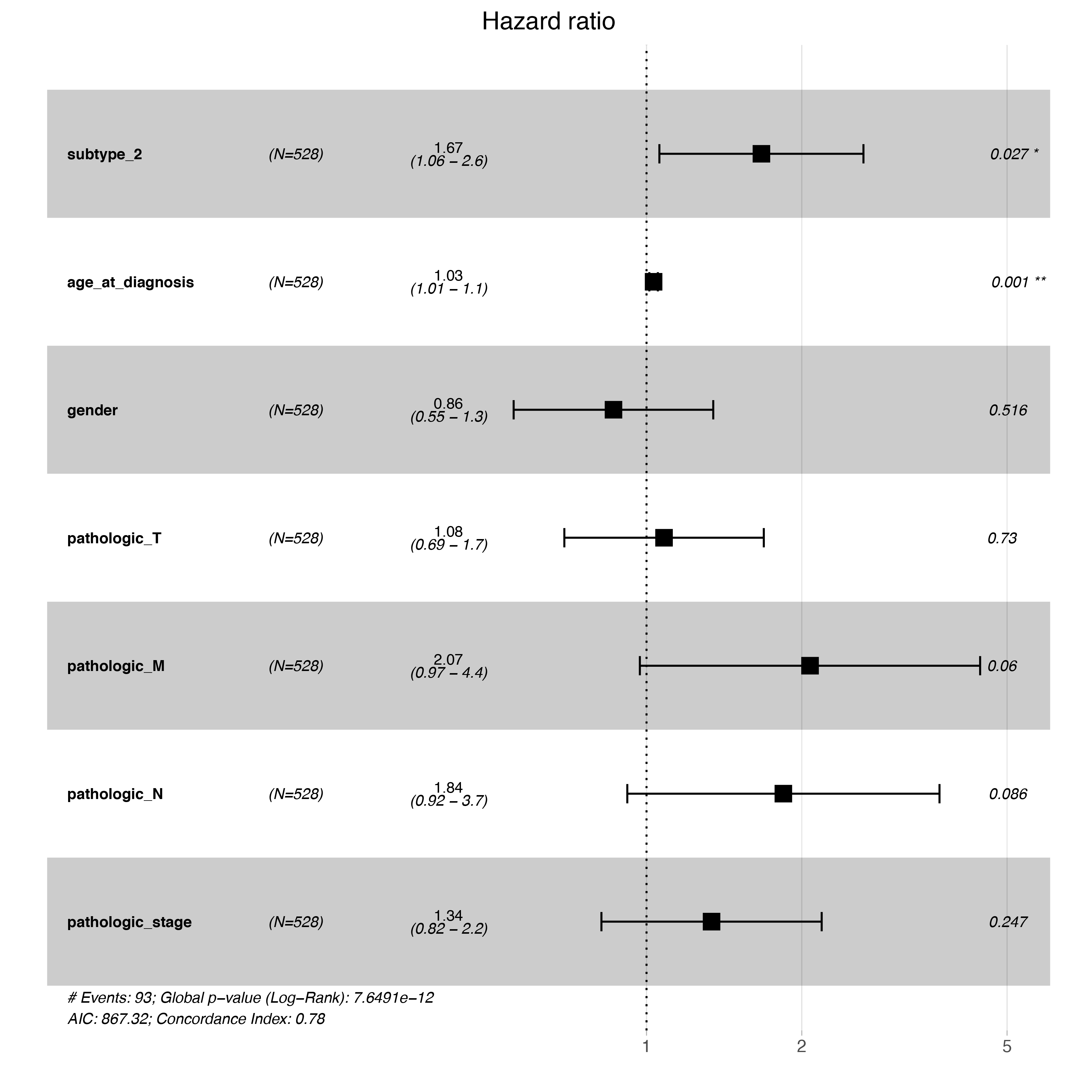

Supplement: Supplementary Figure 2 — Multivariable cox regression. [file Image_2.jpg]
